# Conserved Molecular Signatures in the Spike, Nucleocapsid, and Polymerase Proteins Specific for the Genus *Betacoronavirus* and Its Different Subgenera

**DOI:** 10.3390/genes13030423

**Published:** 2022-02-25

**Authors:** Radhey S. Gupta, Bijendra Khadka

**Affiliations:** 1 Department of Biochemistry and Biomedical Sciences McMaster University, Hamilton, ON L8N 3Z5, Canada; 2 Department of Pharmacology and Toxicology, University of Toronto, Toronto, ON M5S 1A8, Canada; bijendra.khadka@outlook.com

**Keywords:** *Betacoronavirus* and its subgenera *Sarbecovirus*, *Merbecovirus* and *Nobecovirus*, molecular markers, conserved signature indels (CSIs), spike, nucleocapsid and RNA-dependent RNA polymerase (RdRp) proteins, evolution of betacoronaviruses, heptad repeat motifs 1 and 2

## Abstract

The genus *Betacoronavirus*, consisting of four main subgenera (*Embecovirus*, *Merbecovirus*, *Nobecovirus,* and *Sarbecovirus*), encompasses all clinically significant coronaviruses (CoVs), including SARS, MERS, and the SARS-CoV-2 virus responsible for current COVID-19 pandemic. Very few molecular characteristics are known that are specific for the genus *Betacoronavirus* or its different subgenera. In this study, our analyses of the sequences of four essential proteins of CoVs, viz., spike, nucleocapsid, envelope, and RNA-dependent RNA polymerase (RdRp), identified ten novel molecular signatures consisting of conserved signature indels (CSIs) in these proteins which are specific for the genus *Betacoronavirus* or its subgenera. Of these CSIs, two 14-aa-conserved deletions found within the heptad repeat motifs 1 and 2 of the spike protein are specific for all betacoronaviruses, except for their shared presence in the highly infectious avian coronavirus. Six additional CSIs present in the nucleocapsid protein and one CSI in the RdRp protein are distinctive characteristics of either the *Merbecovirus*, *Nobecovirus,* or *Sarbecovirus* subgenera. In addition, a 4-aa insert is present in the spike protein, which is uniquely shared by all viruses from the subgenera *Merbecovirus*, *Nobecovirus,* and *Sarbecovirus*, but absent in *Embecovirus* and all other genera of CoVs. This molecular signature provides evidence that viruses from the three subgenera sharing this CSI are more closely related to each other, and they evolved after the divergence of embecoviruses and other CoVs. As all CSIs specific for different groups of CoVs are flanked by conserved regions, their sequences provide novel means for identifying the above groups of CoVs and for developing novel diagnostic tests. Furthermore, our analyses of the structures of the spike and nucleocapsid proteins show that all identified CSIs are localized in the surface-exposed loops of these protein. It is postulated that these surface loops, through their interactions with other cellular proteins/ligands, play important roles in the biology/pathology of these viruses.

## 1. Introduction

Coronaviruses (CoVs) are a part of the subfamily *Orthocoronavirinae* [1,2]. The members of this family have been divided into four genera, viz., *Alphacoronavirus, Betacoronavirus, Gammacoronavirus*, and *Deltacoronavirus,* based on their branching in phylogenetic trees and genomic structures [1,2,3,4]. Of these four genera, only members of the *Alphacoronavirus* and *Betacoronavirus* genera infect mammals, whereas Gamma- and Delta-CoVs mainly infect birds [1,2]. Of the CoVs infecting humans, Alpha-CoVs causes only mild respiratory diseases, whereas all CoVs causing severe respiratory illnesses in humans, and responsible for different coronaviruses epidemics/pandemics, viz., SARS, MERS, and COVID-19, belong to the genus *Betacoronavirus* [1,2]. The genus *Betacoronavirus* is made up of four main lineages, which are now recognized as distinct subgenera with the names *Embecovirus*, *Merbecovirus*, *Nobecovirus,* and *Sarbecovirus* [1,2,3,4,5]. Of these four subgenera, SARS coronavirus (SARS-CoV) and the COVID-19 virus (named as SARS-CoV-2) are both members of the subgenus *Sarbecovirus*, whereas the MERS-CoV is a part of the *Merbecovirus* subgenus [1,3,5,6]. Thus, in terms of the propensity of CoVs to cause severe diseases in humans, betacoronaviruses and their different subgenera are of central importance. Therefore, it is of much interest to know how the genus *Betacoronavirus* and its different subgenera differ from each other and other CoVs. In this context, it is of much interest to identify molecular markers that are specific for different genera/subgenera of CoVs [1,2,7].

In our recent work, we have reported analyses of the spike (S) and nucleocapsid (N) protein sequences from different sarbecoviruses to identify molecular signatures consisting of conserved signature indels (CSIs) in the S and N proteins, which were specific for different lineages of sarbecoviruses [8]. These studies identified multiple CSIs specific for different lineages of sarbecoviruses including some specific for a cluster consisting of SARS-CoV-2-related CoVs [7,8]. In addition, several signatures were identified, which were specifically shared by other lineages of sarbecoviruses [8]. The molecular markers identified in these studies provided novel means for identifying several distinct clades of sarbecoviruses in molecular terms [7,8]. In addition, the distribution patterns of these molecular signatures in different sarbecoviruses also provided evidence indicating that the SARS-CoV-2 and a pangolin virus (Pangolin CoV-MP_789), whose receptor binding domain is most similar to the SARS-CoV-2 [9,10,11,12], originated by recombination events involving specific sarbecoviruses [8].

Our current understanding of the evolutionary relationships amongst different genera/subgenera of CoVs is primarily based on phylogenetic trees of sequences of the spike and RNA-dependent RNA polymerase (RdRp) proteins [5,11,12,13,14]. In phylogenetic trees, although different genera and subgenera of CoVs form distinct clades, based on these analyses, it remains unclear how different genera and subgenera of CoVs are related to each other [5,11,12,13,14]. Hence, other molecular-sequence-based approaches that can more reliably elucidate the evolutionary relationships among different genera and subgenera of CoVs should be useful in advancing our understanding of these viruses. With this objective, in the present study, we have extended our earlier work on CoVs to identify molecular signatures that are specific for either the entire *Betacoronavirus* genus or those specifically shared by members of different subgenera of *Betacoronavirus*. The conserved signature indels (CSIs) (insertions/deletions) in genes/proteins that are specific for either a given group of organisms/viruses or commonly shared by more than one group/lineage [15,16] provide an important class of molecular markers, that has proven very useful for evolutionary, diagnostic, and taxonomic studies [8,15,17,18,19,20]. The CSIs that are useful for evolutionary studies are generally of specific lengths and are flanked on both sides by conserved regions to ensure their reliability as genetic markers [15,16]. In the present study, we have analyzed the sequences of the spike (S), nucleocapsid (N), envelope (E), and RdRp proteins from different CoVs to look for the presence of CSIs that are specifically shared by either some or all subgenera of *Betacoronavirus*. Results of these studies, which are reported here, have identified ten novel CSIs in the S, N, and RdRp proteins that are either specific for the genus *Betacoronavirus* or one or more of its subgenera, viz., *Merbecovirus*, *Nobecovirus,* and *Sarbecovirus*. The identified molecular markers in addition to providing novel and reliable means for distinguishing the above groups of viruses from each other, as well as all other CoVs, also serve to clarify the evolutionary relationships among different subgenera of *Betacoronavirus*.

## 2. Materials and Methods

### 2.1. Identification of Conserved Signature Indels in Protein Sequences

The identification of CSIs in protein sequences was carried out as described in our recent work [7,8,16]. Briefly, to identify CSIs, sequences for the S, N, E, and RdRp proteins for representative CoVs from different genera and subgenera of CoVs were retrieved from the NCBI database (https://www.ncbi.nlm.nih.gov/genome/, accessed on 30 November 2021) [21] and the GISAID (Global Initiative on Sharing Avian Flu Data) database of SARS CoV-19 sequences [22]. Multiple sequence alignments for these proteins were created using the ClustalW algorithm from the MEGA X (Molecular Analysis Genetic Analysis) software package [23]. These sequence alignments were inspected for any insertion or deletion (indel) in a conserved region specifically present in either all betacoronaviruses or members of its different subgenera. The indels of interest were required to be flanked by at least 4–5 conserved amino acids on each side within the neighbouring 40–50 residues [16,24]. The indels not flanked by conserved regions were not further considered, as they generally do not provide reliable molecular characteristics [16,25]. As the focus of this work was on betacoronaviruses, indels that were specific for other genera of CoVs were not investigated in this study. For the indels that were of interest, query sequences encompassing the conserved indels and their flanking 40–50 amino acids were subjected to a second BLASTp (Basic Local Alignment Search Tool, p refers to protein) search against the NCBI nr (non-redundant) database. All significant hits obtained from these searches were examined to determine the lineage specificities of the identified CSIs. The SIG_CREATE and SIG_STYLE programs described in our earlier work [16] (available on the GLEANS (Gupta Lab Evolutionary Analysis Software), www.gleans.net (accessed on 30 November 2021)) were utilized to create the formatted signature files for different CSIs that are presented here [16]. Sequence information in different figures is shown for only a limited number of viruses (strains) from different genera/subgenera. However, unless otherwise specified, the described CSIs are specific for the indicated lineages, and they are not present in other genera/subgenera of CoVs. Phylogenetic trees based on sequences of the RdRp and spike proteins from representative strains from different lineages of CoVs were constructed using MEGA X as described in our recent work [8].

### 2.2. Analysis of the Available Protein Structures to Map the Structural Locations of CSIs

The structural locations of the identified CSIs were mapped in the spike and nucleocapsid (N) protein structures using the experimentally solved three-dimensional (3D) structures obtained from the Protein Data Bank (PDB) [26]. The superimpositions of the 3D structures were carried out using PyMOL (Version 1.7.4; Schrödinger, LLC, (New York, NY, USA) to examine the structure, features, and location of identified CSIs in the spike and N-protein structure. In the absence of experimentally solved structures, computational techniques such as comparative protein structure modelling or homology modelling can be utilized to generate the 3D structure of target proteins [27]. Use of homology models to analyze the structural locations of CSIs has been described in several of our previously published works [28,29,30,31].

## 3. Results

### 3.1. Phylogenetic Relationships among Coronaviruses

**Figure 1:**
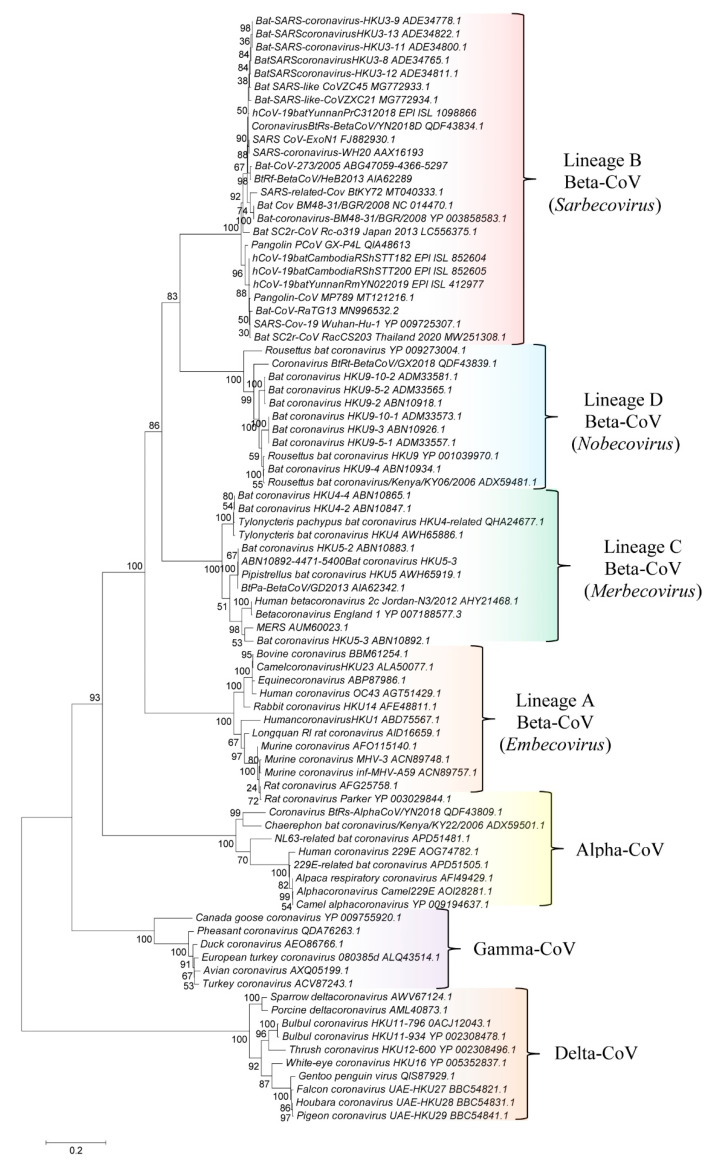
A maximum-likelihood distance tree based on sequence alignment of the RNA-dependent RNA polymerase (RdRp) protein from representative viruses/strains from different genera/subgenera of CoVs. The tree was bootstrapped 100 times and the % bootstraps for different branches are indicated on the nodes. The clades corresponding to different genera and subgenera within the genus *Betacoronavirus* are labeled.

Figure 1 shows a phylogenetic tree based on the sequences for RdRp protein from representative CoVs from the subfamily *Orthocoronavirinae*. In accordance with earlier studies [1,2,3], members of the four *Orthocoronavirinae* genera, viz., *Alphacoronavirus, Betacoronavirus, Gammacoronavirus,* and *Deltacoronavirus,* form distinct clades in this tree. Additionally, within the genus *Betacoronavirus*, four main clusters corresponding to its four subgenera i.e., *Embecovirus*, *Sarbecovirus*, *Merbecovirus,* and *Nobecovirus* are also observed. These clusters are marked in the tree along with their commonly known clade designations (i.e., clades A, B, C, and D). Similar branching of the *Orthocoronavirinae* viruses is seen in a phylogenetic tree based on the spike protein (Supplementary Figure S1) and in earlier phylogenetic studies based on spike and RdRp proteins [1,2,3]. The trees shown in Figure 1 and Figure S1 provide a phylogenetic framework to understand the significance of various identified molecular signatures.

### 3.2. Molecular Markers (CSIs) Specific for the Genus Betacoronavirus and Its Different Subgenera

The main objective of this work was to identify molecular signatures (CSIs) in the S, N, E, and RdRp proteins that are specific for the genus *Betacoronavirus* or its different subgenera. These studies have identified several novel CSIs in the S, N, and RdRp proteins. However, no useful CSI was detected in the envelope protein. We discuss below the group specificity and characteristics of the identified CSIs.

**Figure 2:**
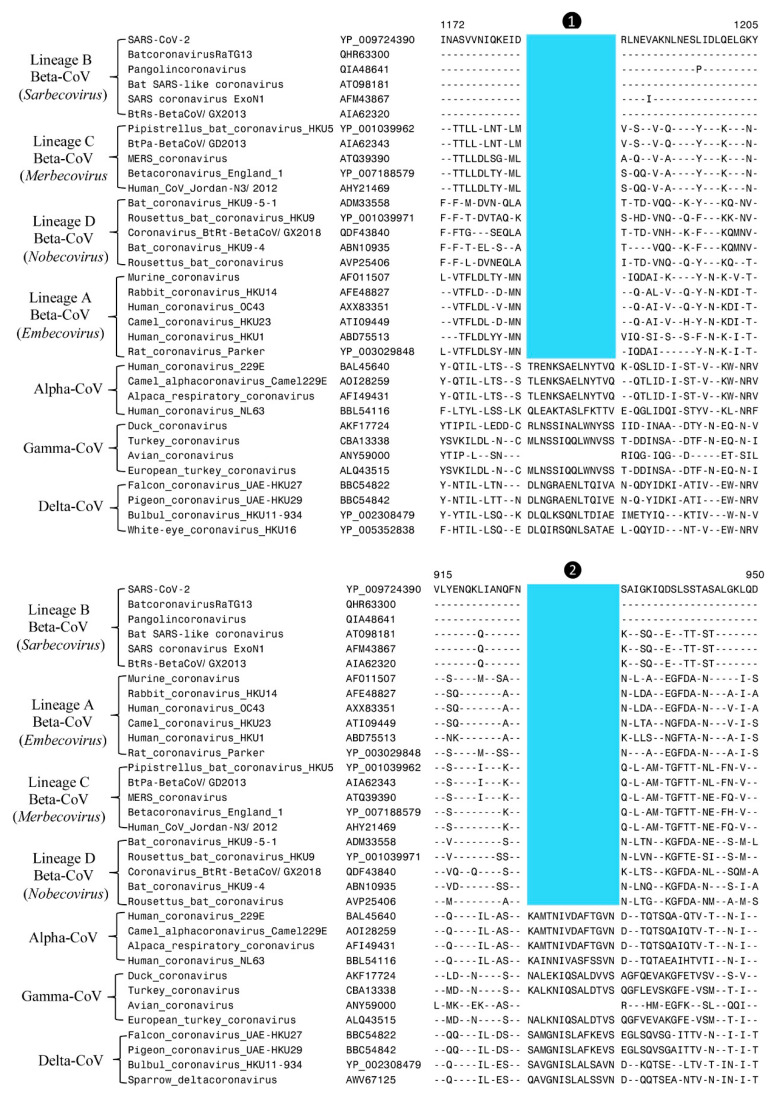
Partial sequence alignments of two conserved regions from the spike protein showing two different CSIs that are specific for the genus *Betacoronavirus*. The CSIs present in these sequence alignments are highlighted in blue and they are labeled ❶ and ❷. Both CSIs are commonly shared by all members of the genus *Betacoronavirus*, but barring one exception, avian coronavirus, they are not found in any other CoV. Dashes (–) in these and all other sequence alignments denote identity with the amino acid shown in the top sequence. The numbers on the top indicate the locations of these sequence regions within the indicated proteins. The accession numbers of different proteins are given in the second column.

Our analyses have identified two large CSIs in the spike protein that are commonly shared by all betacoronaviruses. Figure 2 shows partial sequence alignments of two different conserved regions from the S2 subunit of the spike protein where these CSIs are found. The CSIs in these sequence alignments are colour-highlighted and, in both cases, they consist of 14-aa deletions within conserved regions of the spike protein. The dashes (-) in these alignments indicate identity with the amino acid on the top line. Sequence information in Figure 2 is shown for only a limited number of viral strains from different genera and subgenera of CoVs, however all other members of the indicated genera/subgenera also contained or lacked the indicated CSIs. As seen from Figure 2, both these CSIs (marked ❶ and ❷) are present in the spike protein homologs from different subgenera of betacoronaviruses but barring one exception they are not found in any other genera of CoVs. The Omicron variant of SARS-CoV-2 contains large numbers of changes in the spike protein including several insertions and deletions [32]. However, in the sequence region where these two CSIs are found, no changes are observed in the omicron variant (results not shown). Besides the betacoronaviruses, the only other virus which contains these two CSIs is avian coronavirus belonging to the genus *Gammacoronavirus* (see Figure 1 and Figure S1). However, other viruses from this genus do not contain these CSIs. The most likely explanation to account for the distribution of these two CSIs in different CoVs is that the genetic changes giving rise to these CSIs occurred in a common ancestor of the betacoronaviruses, and these changes were then retained by all members of this genus. The presence of these two CSIs in the avian coronavirus can result from either a genetic recombination with a betacoronavirus [33] or by means of independent occurrence of these genetic changes in this virus.

#### Molecular Markers (CSIs) Specific for Different Groups (Subgenera) of *Betacoronavirus*

**Figure 3:**
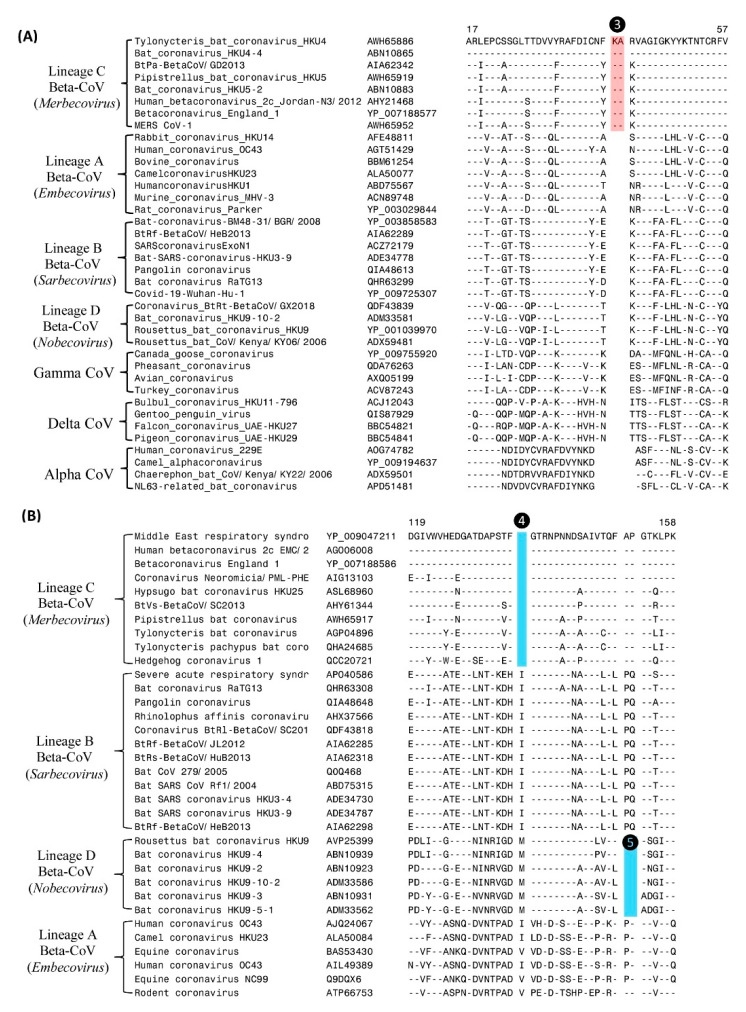
Partial sequence alignments of two conserved regions from the RdRp and nucleocapsid proteins showing a number of CSIs that are specific for the subgenera *Merbecovirus* and *Nobecovirus*. (**A**) Partial sequence alignment of RdRp protein showing a CSI consisting of 2-aa insertion (highlighted in blue and labeled ❸) which is specific for the *Merbecovirus*. (**B**) Partial sequence alignment of nucleocapsid protein showing two different CSIs, one of which (❹) is specific for the subgenus *Merbecovirus* and another CSI (labeled ❺), which is only present in different viruses from the subgenus *Nobecovirus*.

The subgenus *Merbecovirus* of *Betacoronavirus* includes the virus responsible for the Middle East respiratory syndrome (MERS) [1,2]. Although the viruses from this subgenus form a distinct clade in phylogenetic trees (see Figure 1), there is no known molecular signature that is specific for this group of CoVs. Our analyses have identified two CSIs, in the RdRp and N proteins, that are uniquely shared by different members of this subgenus. Partial sequence alignments of the RdRp and N proteins showing the CSIs that are specific for the subgenus *Merbecovirus* are presented in Figure 3.

In the sequence alignment of RdRp protein shown in Figure 3A, a 2-aa insertion (marked ❸) is present in a conserved region (highlighted in cyan) that is commonly shared by all merbecoviruses, but it is absent in all other betacoronaviruses as well other genera of CoVs. Likewise, in the partial sequence alignment of the N-protein presented in Figure 3B, a 1-aa deletion (marked ❹) is present, which is again a unique characteristic of the members of the subgenus *Merbecovirus*. The genetic changes responsible for these CSIs are postulated to have occurred in a common ancestor of the subgenus *Merbecovirus*, and they provide novel and reliable means for distinguishing members of this subgenus from all other CoVs.

**Figure 4:**
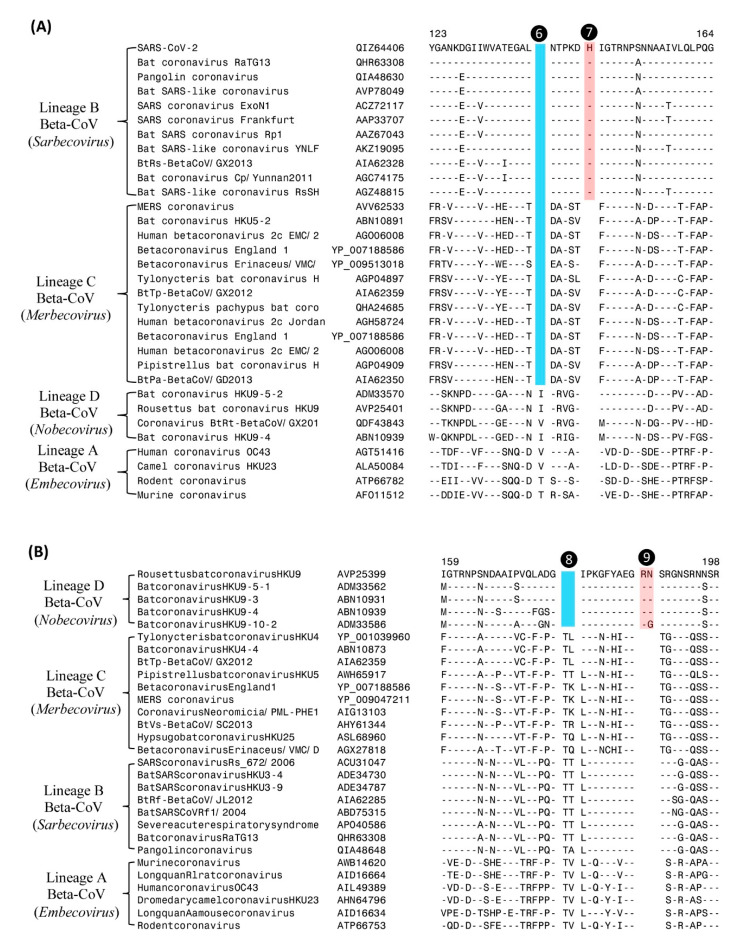
Partial sequence alignments of two conserved regions of the nucleocapsid proteins showing a number of CSIs specific for different subgenera of *Betacoronavirus*. (**A**) This sequence region depicts two different CSIs. The CSI labeled ❻ is commonly shared by different viruses from the subgenera *Merbecovirus* and *Sarbecovirus*, whereas the CSIs marked ❼ is specific for the viruses from the subgenera *Sarbecovirus*. (**B**) This sequence region depicts two CSIs marked ❽ and ❾ which are specific for the subgenus *Nobecovirus*. Dashes (–) in the alignments indicate identity with the amino acid shown in the top sequence.

In the sequence alignment of the N-protein shown in Figure 3B, in addition to the CSI that is specific for *Merbecovirus*, there is another 2-aa deletion (marked ❺) present, which is specific for the subgenus *Nobecovirus*. In Figure 4, we present sequence alignments of two other conserved regions of the N-protein, where multiple CSIs specific for different lineages of *Betacoronavirus* are found. In the sequence alignment shown in Figure 4A, a CSI consisting of a 1-aa deletion is present (marked ❻) that is commonly shared by all viruses from the subgenera *Merbecovirus* and *Sarbecovirus*, but not found in the other two subgenera of *Betacoronavirus*. Close to this CSI, there is another CSI present consisting of a 1-aa insertion (marked ❼), which is uniquely shared by all viruses from the subgenus *Sarbecovirus*. Both these CSIs are separated by conserved regions indicating that they constitute reliable characteristics and are not caused by sequence alignment artifacts. Figure 4B shows two additional CSIs, one consisting of a 2-aa deletion (marked ❽) and another consisting of a 2-aa insertion (marked ❾), which are also specific for the CoVs from the subgenus *Nobecovirus*. Based on these CSIs, members of the subgenera *Nobecovirus* and *Sarbecovirus* can be reliably distinguished from other CoVs.

**Figure 5:**
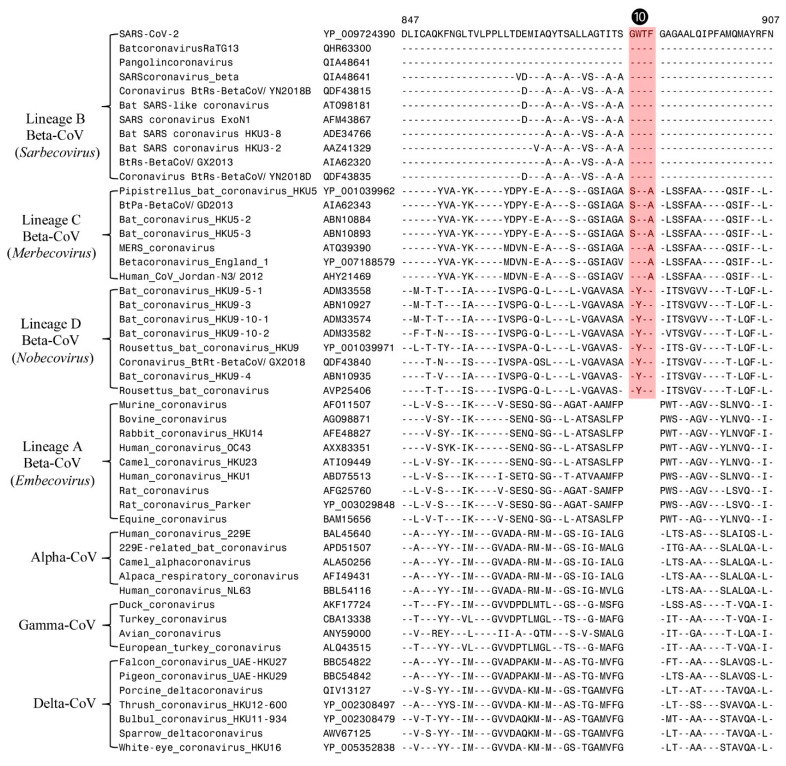
Excerpts from the sequence alignment of the spike protein showing a 4-aa CSI that is present only in the *Betacoronavirus* subgenera *Merbecovirus, Nobecovirus*, and *Sarbecovirus***.** This CSI (❿) provides evidence that the CoVs from these subgenera are more closely related to each other, and they evolved after the divergence of other CoVs. No change is observed in this region in the Omicron variant.

Lastly, in Figure 5 we present partial sequence alignment of the spike protein, where a 4-aa insertion (marked ❿) in a conserved region is commonly shared by all CoVs from the subgenera *Merbecovirus, Nobecovirus*, and *Sarbecovirus*. This insert is absent in all viruses from the subgenus *Embecovirus* as well as by viruses from other CoVs genera, viz., Alpha-, Delta-, and Gamma-CoVs). Based on its distribution in different CoVs, the genetic change giving rise to this CSI is postulated to have occurred in a common ancestor of the subgenera *Merbecovirus, Nobecovirus*, and *Sarbecovirus* after the divergence of *Embecovirus* as well as other genera of CoVs.

### 3.3. Localizations of the CSIs in Protein Structures

**Figure 6:**
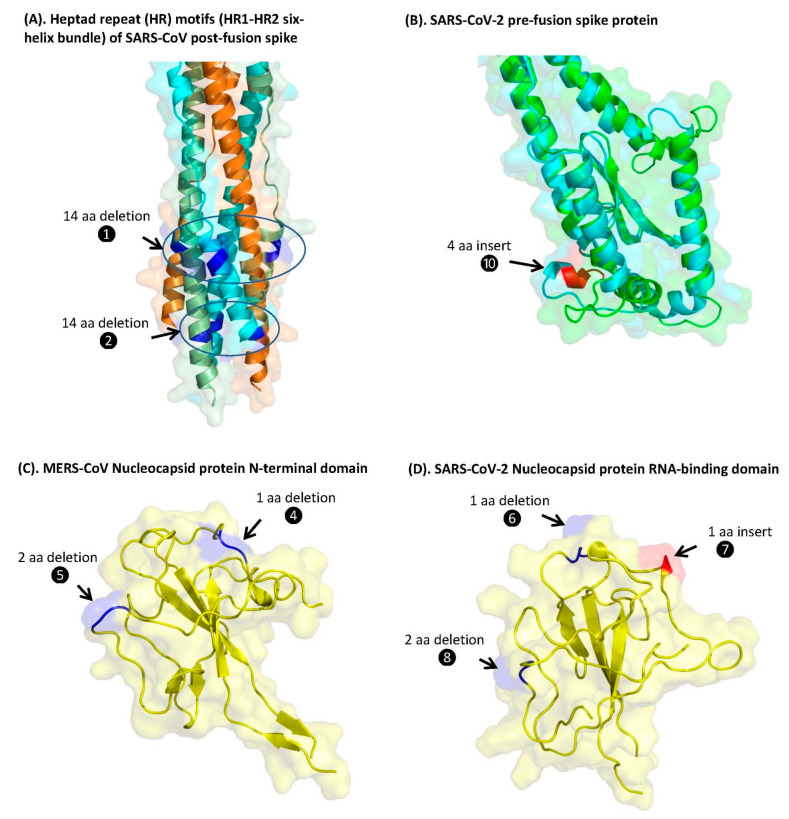
Mapping the surface locations of eight of the identified CSIs in the spike and nucleocapsid proteins. (**A**) Cryo-EM-based structure of the post-fusion form of the SARS-CoV spike protein (PDB ID: 6m3w) showing the structural location of CSIs ❶ and ❷. The regions where these CSIs are found are circled. Of these two CSIs, (❶) is present within the HR2 motif, whereas CSI ❷ is found within the HR1 motif in the S2 subunit. (**B**) The structural location of the 4-aa CSI (❿), which is commonly shared by the *Merbecovirus, Nobecovirus*, and *Sarbecovirus* subgenera, using a superimposed structure of the spike protein from SARS-CoV-2 (shown in green) and the PEDV virus (shown in cyan color). (**C**) Crystal structure of the N-terminal domain of the N-protein (PDB ID: 6LNN) from MERS-CoV in which the structural locations of two CSIs (❹ and ❺) are highlighted. (**D**) Structure of the RNA-binding domain (RBD) of the N-protein (PDB ID: 7R98) from SARS-CoV-2 depicting the structural locations of three CSIs (❻, ❼, and ❽) in RBD.

We have also mapped the locations of eight of the identified CSIs in the spike and N-proteins using their 3D structural coordinates (Figure 5). For these studies, we have used the available structures of the spike protein from SARS-CoV-2 (PDB ID: 6VSB) [34] in both pre-fusion and post-fusion state and the cryo-EM based structure of the porcine epidemic diarrhea virus (PEDV) (PDB ID: 6U7K_A) [35], which is an alphacoronavirus. The CSIs in the spike protein for which the structural localization was determined include two large 14-aa deletions (❶ and ❷) which are specific for the genus *Betacoronavirus*, and a 4-aa CSI (❿) which is commonly shared by members of the subgenera *Merbecovirus, Nobecovirus*, and *Sarbecovirus* (Figure 6). 

The cartoon representations of the superimposed forms of the 3D structures of the spike proteins from CoVs containing these CSIs are presented in Figure 6. In panel A of Figure 6, a cryo-EM-based structure of the post-fusion form of the SARS-CoV spike protein (PDB ID: 6m3w) was utilized to show the structural location of two large 14-aa CSIs (❶ and ❷). Of these CSIs, CSI ❶ is present within the conserved heptad repeat 2 (HR2) motifs, and CSI ❷ is present within the heptad repeat 1 (HR1) motif in the S2 subunit of the spike protein. Both the HR1 and HR2 motifs, which form a six-helical bundle in the S2-subunit, play a key role in mediating fusion and entry of CoV-2 into the host cell [36,37]. In Figure 6B, the structural location of the 4-aa CSI (❿ in Figure 5), which is commonly shared by the *Merbecovirus, Nobecovirus,* and *Sarbecovirus* subgenera, is shown using a superimposed structure of the spike proteins from SARS-CoV-2 (shown in green) and the PEDV-virus (shown in cyan color). In panel C, we show a crystal structure of the N-terminal domain of the N-protein (PDB ID: 6LNN) from MERS-CoV in which the structural locations of two CSIs (❹ and ❺) are highlighted. Similarly in panel D, we show the structure of the N-protein RNA-binding domain (RBD) (PDB ID: 7R98) from SARS-CoV-2 to depict the structural locations of three CSIs (❻, ❼, and ❽ shown in Figure 4A,B). As seen from these figures, all the CSIs identified and analyzed in this study are present in the surface-exposed loop regions of the spike and N-protein structures. 

The structural localization of the two other CSIs i.e., a 2-aa insert (❸ shown in Figure 3A) present near the N-terminal end of the RdRp protein, and a 2-aa insert (❾ shown in Figure 4B) which is present in the RBD of N-protein, were not determined in this study, as experimentally solved structural information for these sequence regions is not available.

## 4. Discussion

CoVs have been responsible for three major outbreaks in the past 20 years including the current COVID-19 pandemic caused by the SARS-CoV-2 virus, which has infected >307 million people worldwide leading to >5.6 million deaths (https://coronavirus.jhu.edu/ (accessed on 2 February 2022) [1,2,5,6,38,39,40,41]. The two earlier outbreaks of CoVs, known as the severe acute respiratory syndrome (SARS) and the Middle East respiratory syndrome (MERS), were caused by the SARS-CoV and MERS-CoV, respectively. Although the CoVs are comprised of four genera, all CoVs responsible for the major outbreaks/pandemics are a part of the genus *Betacoronavirus*. Of the four main *Betacoronavirus* subgenera, both SARS-CoV and SARS-CoV-2 are part of the *Sarbecovirus* subgenus, whereas the MERS-CoV belongs to the subgenus *Merbecovirus* [1,3,5,6]. Thus, in terms of the clinical significance and human health impact, members of the genus *Betacoronavirus* are of utmost importance. Other viruses such as OC43, and HKU1, which cause mild common cold-like symptoms, are a part of the *Embecovirus* subgenus [1]. In this study, we analyzed the sequences of four major conserved structural proteins i.e., spike, nucleocapsid, envelope, and RdRp proteins, which play central roles in cellular infection and replication [1,2], for the presence of conserved signature indels that are either specific for the genus *Betacoronavirus* or its constituent subgenera. The results of these studies have identified ten novel CSIs and information regarding the viral group specificity and some characteristics of these CSIs are summarized in Table 1. 

Of these CSIs, two CSIs in the spike protein are specific for all members of the genus *Betacoronavirus*, whereas six CSIs found in the N-protein and one CSI found in RdRp protein are specific for members of the betacoronaviruses’ subgenera *Sarbecovirus, Merbecovirus*, and *Nobecovirus*. One additional identified CSI in the spike protein (CSI ❿, Figure 5) is shared explicitly by viruses from the *Merbecovirus, Nobecovirus*, and *Sarbecovirus* subgenera, providing insights into the branching order and evolutionary relationships among the *Betacoronavirus* lineages. The distribution pattern of this CSI provides evidence that the CoVs from these three subgenera are more closely related to each other, and they evolved after the divergence of the embecoviruses and viruses from other CoVs genera, that do not contain this CSI. It is of interest that viruses from the subgenus *Embecovirus*, which lack this CSI, have been reported to differ from the other three betacoronavirus subgenera in that they contain an additional shorter spike-like protein, hemagglutinin esterase [42], which is not present in the three *Betacoronavirus* subgenera containing this 4-aa insert in the spike protein. Although the presence of this 4-aa insert coincides with the loss of the hemagglutinin esterase protein from these three subgenera, it is unclear whether these two genetic events are functionally correlated. The shared presence of the CSIs ❶, ❷, and ❿ by a number of different subgenera of betacoronaviruses indicates that they represent important conserved properties of these viruses, while these viruses differ from each other in other regards.

Earlier work on the CSIs shows that the genetic changes represented by them are functionally important for the group of organisms for which these CSIs are specific [30,43]. Furthermore, earlier studies showed that all studied CSIs are localized in surface-exposed loops of the structures of different proteins [19,28,29,30,31,44]. In accordance with the results from earlier studies, all eight CSIs whose structural localization was analyzed in this study were also found to be located within the surface-exposed loops of the spike and nucleocapsid proteins. Surface-exposed loops in proteins are known to play important functional roles by mediating novel protein–protein or protein–ligand interactions [29,43,45,46]. Of the CSIs identified in the present work, the two CSIs, which are specific for the genus *Betacoronavirus*, are both comprised of 14-aa deletions in the spike protein. Interestingly, one of these CSIs (❷) is present within conserved heptad repeat 1 (HR1) motif, whereas the other CSI (❶) is found within the heptad repeat 2 (HR2) motif of the spike protein S2 subunit. The HR1 and HR2 motifs in the S2 subunit are known to interact with each other to form a six-helical bundle, which by bringing viral and cellular membranes in proximity, plays a crucial role in mediating membrane-fusion and entry of CoV-2 into the host cell [36,37,47]. As both these large CSIs are deletions, in other genera of CoVs, that do not contain these deletions, the lengths of the HR1 and HR2 motifs are longer than those found in the betacoronaviruses. It is of much interest to note that these two large CSIs, in addition to the members of the genus *Betacoronavirus* are also commonly shared by the avian coronavirus, which is a *Gammacoronavirus*. The avian CoV, also known as infectious bronchitis virus (IBV) is a highly infectious virus of major economic concern and is responsible for most of the infections caused by the Gammacoronaviruses [48,49]. It is unclear at present, how these important changes brought about by these two large CSIs may affect the cellular function of the spike protein in the betacoronaviruses and avian-CoV. However, in view of the fact that the viruses containing these large deletions (CSIs) comprise the most infectious members of the Orthocoronavirinae family, it is hypothesized that these genetic changes likely play an important role in determining the pathogenicity and infectiousness of the coronaviruses.

With regard to the functional significance of the CSIs, it should be noted that two of the CSIs in the spike protein described in our recent work, which are commonly shared by both SARS-CoV-2r cluster of viruses and the SARS viruses [7,12,50], are located within the receptor-binding domain of the spike protein and their sequences form a significant portion of the receptor-binding motif of these viruses [5,51,52,53,54]. The residues from these CSIs have been shown to play a critical role in the binding of spike protein from these viruses to the human ACE2 receptor [12,13,51,53,55,56]. These observations underscore the importance of the identified CSIs in the functioning and pathogenicity of viruses. Although the functions of the CSIs identified in the present work, which are specific for *Betacoronavirus* or its different subgenera are presently not known, based on earlier work, it is hypothesized that these CSIs will also be playing important roles in the functioning of these CoVs. Thus, it should be of interest to examine the functional roles of these CSIs by experimental studies. 

In addition to the CSIs identified in the present work, our recent work also identified several CSIs specific for several lineages of *Sarbecovirus*, including two CSIs specific for the SARS-CoV-2-related cluster of viruses [7]. Because of the specificities of the CSIs identified in the present study and our earlier work for different lineages of betacoronaviruses, they provide novel molecular means for distinguishing viruses from these groups/lineages from each other. Furthermore, as all CSIs identified in this study, and in our earlier work [8], are flanked by conserved regions, the sequences for these regions provide potential means for developing novel diagnostic tests to identify these specific viruses [57,58]. These tests can be based on different commonly employed experimental techniques, viz., PCR-based, q-PCR-based, immunological, or antibody-based methods, as well as in silico identification in genomic and metagenomic sequences by means of BLAST searches. It should be noted that the CSIs have been successfully used previously for development of highly specific diagnostic tests for two important bacterial pathogens [16,57,58].

## Figures and Tables

**Table 1:** Conserved signature indels found in spike, nucleocapsid, and RNA-dependent RNA polymerase (RdRp) proteins that are specific for various members of lineages of coronaviruses.

Protein Name	Acc. No:	Indel Length	Indel Location	Indel Specificity	Figure No:
Spike	YP_009724390	14-aa del	1172–1205	β-CoV	Figure 2
Spike	YP_009724390	14-aa del	915–950	β-CoV	Figure 2
RdRp	AWH65886	2-aa ins	17–57	*Merbecovirus*	Figure 3A
Nucleocapsid	YP_009047211	1-aa del	119–158	*Merbecovirus*	Figure 3B
Nucleocapsid	YP_009047211	2-aa del	119–158	*Nobecovirus*	Figure 3B
Nucleocapsid	QIZ64406	2-aa del	123–169	*Sarbecovirus* and *Merbecovirus*	Figure 4A
Nucleocapsid	QIZ64406	1-aa ins	123–169	*Sarbecovirus*	Figure 4A
Nucleocapsid	AVP25399	2-aa del	159–198	*Nobecovirus*	Figure 4B
Nucleocapsid	AVP25399	2-aa ins	159–198	*Nobecovirus*	Figure 4B
Spike	YP_009724390	4-aa ins	847–907	*Sarbecovirus*, *Merbecovirus* and *Nobecovirus*	Figure 5

## Data Availability

The data presented in this study are available in publicly accessible repository (https://www.ncbi.nlm.nih.gov/genome/, accessed on 30 November 2021) and supplementary material here.

## Supplementary Materials

The following are available online at https://www.mdpi.com/article/10.3390/genes13030423/s1. Figure S1. A maximum-likelihood tree based on the sequence alignment of spike protein from representative viruses/strains from different genera/subgenera of CoVs.
