# Supplementary material for: Conserved Molecular Signatures in the Spike, Nucleocapsid, and Polymerase Proteins Specific for the Genus Betacoronavirus and Its Different Subgenera
Source: Genes (Basel). 2022 Feb 25;13(3):423. doi: 10.3390/genes13030423 (PMC8949385; doi:10.3390/genes13030423)
Supplement: Supplementary file 1 [file genes-13-00423-s001.zip › genes-1605870-supplementary.pdf]

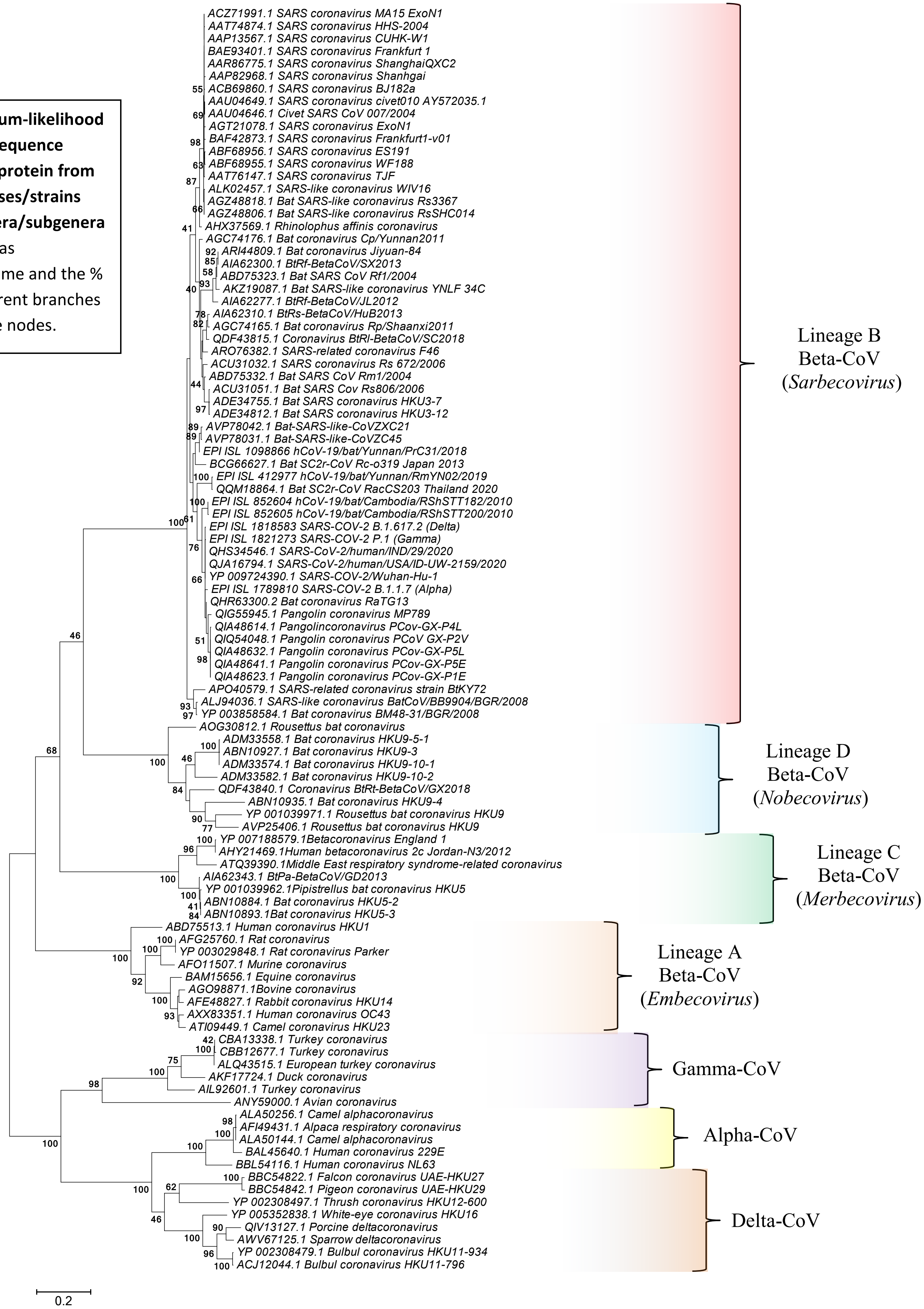

Figure S1. A maximum-likelihood tree based on the sequence alignment of spike protein from representative viruses/strains from different genera/subgenera of CoVs. The tree was bootstrapped 100 time and the % bootstraps for different branches are indicated on the nodes.
